# Supplementary material for: The Weak Relationship between Vitamin D Compounds and Glucose Homeostasis Measures in Pregnant Women with Obesity: An Exploratory Sub-Analysis of the DALI Study
Source: Nutrients. 2022 Aug 9;14(16):3256. doi: 10.3390/nu14163256 (PMC9415540; doi:10.3390/nu14163256)
Supplement: Supplementary file 1 [file nutrients-14-03256-s001.zip › nutrients-1806379-supplementary.pdf]

Supplementary Table S1. Associations between vitamin D compounds and **pregnancy-validated** glucose homeostasis variables **according to 25OHD sufficiency**, in a multivariate model adjusted for age, body mass index, ethnicity, family history of diabetes, prior GDM, recruitment site and (at 24-28 and 35-37 wks) DALI lifestyle intervention.

Outcome variables	***β*** values/OR for significant associations								
	<20 weeks			24-28 weeks			35-37 weeks		
25OHD (mmol/l)	<30	30-50	≥50	<30	30-50	≥50	<30	30-50	≥50
N of women	97	210	605	57	127	476	52	102	348
**Fasting**									
FPG			0.101* (D3)		-0.255* (D2)				
1/HOMA-IR (sens)									0.170* (D3)
QUICKI (sens)									0.152* (D3)
HOMA-β (sec)								0.287** (D3)	
Fasting DI									
• QUICKI* HOMA-β								0.298** (D3)	
• 1/HOMA-IR* HOMA-β									
**Post-challenge**									
1h PG		0.204* (D3)		0.302* (EPI)		0.139* (D3)			
2h PG		0.390** (D3)			-0.341** (D3)				
Matsuda (sens)	0.462** (EPI)	-0.348** (D3)							0.238* (D3)
Stumvoll phase 1 (sec)							0.365* (D2)		
$AUC_{ins/glu}$ (sec)									
Post-challenge DI									
• Matsuda*AUC		0.352** (D2)	-0.175* (EPI)					0.383* (D3)	0.266* (D2) 0.255* (D3)
• Matsuda*Stumvoll1	0.412* (EPI)	-0.331* (D3)	-0.190* (EPI)						
HiP		1.134*¶ (D3)							

*=p<0.05, **=p<0.01 ¶ OR

D2: 25OHD2, D3: 25OHD3, EPI: C3-epimer, FPG: fasting plasma glucose, HOMA-IR: homeostasis model assessment insulin resistance, sens: insulin sensitivity index, QUICKI: quantitative insulin sensitivity check index, HOMA- β: homeostasis model assessment beta, sec: insulin secretion index; DI: disposition index, PG: plasma glucose, $AUC_{ins/glu}$: area under the curve insulin/glucose, HiP: Hyperglycemia in pregnancy.
